# Unpacking Protection Motivation Theory: evidence for a separate protective and non-protective route in private flood mitigation behavior

**DOI:** 10.1080/13669877.2018.1485175

**Published:** 2019-01-14

**Authors:** Philipp Babcicky, Sebastian Seebauer

**Affiliations:** a Wegener Center for Climate and Global Change, University of Graz, Graz, Austria;; b LIFE—Centre for Climate, Energy and Society, Joanneum Research Forschungsgesellschaft mbH, Graz, Austria

**Keywords:** Flood risk management, risk research, climate change adaptation, risk perception, coping beliefs, household flood preparedness

## Abstract

Flood preparedness of private households is regarded an essential building block of integrated flood risk management. In the past decade, numerous empirical studies have employed the protection motivation theory (PMT) to explain flood mitigation behavior at the household level. However, much of this research has produced mixed results and could not consistently confirm the strength and direction of the relationships between the PMT components. Based on a survey of 2,007 households in flood-prone areas, this study revisits the model structure of the PMT by means of structural equation modeling. Compared to the methods used in previous studies, this modeling technique allows us to capture the PMT components in greater detail and to comprehensively test their hypothesized interrelations. Our results point to two separate routes leading to two different response types: A protective route from coping appraisal to protective behavior, and a non-protective route from threat appraisal to non-protective responses. Risk perception is not found to be part of the protective route, neither are non-protective responses confirmed to undermine protection motivation. The two separate routes are observed consistently across all combinations of the six protective and four non-protective responses assessed in this study. In the light of encouraging private flood adaptation, risk communication measures should specifically target the protective route and avoid (accidentally) providing incentives that fall within the non-protective route. This cross-sectional study, however, cannot establish how the two routes interrelate over time. More experimental and longitudinal research is required to address potential feedback effects and the role of decision stages.

## 1. Introduction

Precautionary measures at the household level are regarded as an essential component of integrated flood risk management (Bubeck et al. 2013). The responsibility of flood-prone residents to take action has been emphasized in both regional (EU 2007) and national flood policies (e.g. BMLFUW 2017). Indeed, recent research has shown that private measures, such as installing flood barriers, adapting furniture or raising electrical appliances, can considerably reduce flood damage (Poussin, Botzen, and Aerts 2015; Botzen, Aerts, and van den Bergh 2009; Kreibich et al. 2005). However, numerous households in flood-prone areas are still hesitant to engage in flood mitigation (Bubeck, Botzen, and Aerts 2012). Given this lack of action, several attempts have been made to explain, as Grothmann and Reusswig (2006, 101) put it succinctly, ‘why some residents take precautionary action while others do not’.

Systematic research in understanding private flood mitigation behavior benefits from coherent theoretical frameworks that allow for comparison and generalization of findings across different studies, as opposed to the nontheoretical and exploratory approaches that have been dominating the literature (Kellens, Terpstra, and De Maeyer 2013). Against this background, Grothmann and Reusswig (2006) introduced the protection motivation theory (PMT) to flood risk research more than a decade ago. The PMT, originally developed in health psychology, suggests that the motivation to protect from a specific threat depends on how a person balances threat appraisal against coping appraisal (Rogers 1983). Subsequently, an increasing number of studies have applied the PMT as a theoretical framework to explain protective behavior of citizens at risk from flooding (see Section 2.1).

Now, a decade after Grothmann and Reusswig’s seminal work and after a substantial body of literature on flood mitigation behavior has accumulated, this article revisits the model structure of the PMT. Building on both a review of pertinent studies and an analysis of a large-scale survey in Austria, we suggest that certain decision paths underlying the PMT are stronger and more consistent than others. Our results indicate that when applying the PMT in the flood risk domain, two separate paths emerge that lead two to distinct endpoints (i.e. response types): a *protective route*, from coping appraisal to protective behavior, and a *non-protective route*, from threat appraisal to non-protective responses. Expanding on the methodology used in previous studies, the statistical technique of structural equation modeling (SEM) enables us to capture the PMT components in their full granularity and to comprehensively test their expected interrelations. Our results have important implications for flood risk communication, for example, that promoting risk perception should not be expected to lead to protective behavior.

This article begins by giving a brief overview of the PMT and compiling findings that indicate the presence of two distinct routes within the PMT framework. Section 3 describes the study design and operationalization of the theoretical model. Model results are presented, discussed and related to insights from previous studies in Section 4. Finally, the article concludes with implications for policy-making, limitations, and suggestions for future research in Section 5.

## 2. Explaining private flood mitigation behavior

### 2.1. The structure of the PMT

The PMT originates from health psychology and was initially developed to explain the effect of fear appeals on health-related behavior (Rogers 1983, 1975). Over the past, the PMT and selected key components have also been applied in research on natural and environmental hazards, including droughts (Truelove, Carrico, and Thabrew 2015), earthquakes (Mulilis and Lippa 1990), volcanic hazards (Paton, Smith, and Johnston 2000), tornados (Weinstein et al. 2000), wildfires (Martin, Bender, and Raish 2008), and flood risks (Richert, Erdlenbruch, and Figuières 2017; Dittrich et al. 2016; Le Dang et al. 2014; Zaalberg et al. 2009; Grothmann and Reusswig 2006; to name a few).

**Figure 1.**
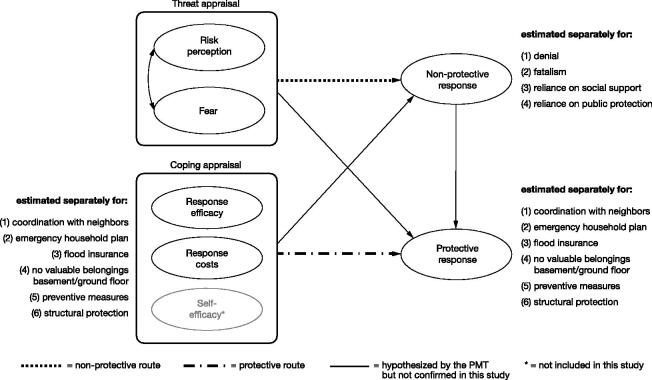
The structural model according to the PMT.

The PMT proposes a clear model structure mapping out the components and processes of protective behavior (see Figure 1). In his revised version of the PMT, Rogers (1983) posits two cognitive processes that determine changes in coping intentions, which are expected to translate into coping actions. These two processes are referred to as *threat appraisal* and *coping appraisal*. The PMT states that the two processes are executed sequentially: Once a certain level of threat appraisal is exceeded, a person starts to evaluate its coping options. Rogers (1983) points to a potential interaction between the two processes: High threat appraisal together with high coping appraisal is suggested to lead to protection motivation. If high threat appraisal meets low coping appraisal, however, protection motivation remains low. In a later study, Rippetoe and Rogers (1987) introduce *protective* and *non-protective* response types. Whereas the former reduces the physical risks of a specific threat, the latter response type (e.g. denial, fatalism, and avoidance) reduces solely the emotional consequences of the threat. Rippetoe and Rogers (1987) emphasize that the distinction between protective and non-protective responses is essential since they may have different antecedents (e.g. one response type might be closer related to threat appraisal, the other one closer to coping appraisal).

The following section provides a detailed account of the PMT components and their interrelations as reported in previous studies on private flood mitigation.

### 2.2. Previous findings on the relationships between PMT components

#### 2.2.1. Threat appraisal

Threat appraisal captures how threatened a person feels by a certain risk (Bubeck, Botzen, and Aerts 2012) and is composed of a cognitive (risk perception) subcomponent and an affective one (threat-related feelings). In the context of flooding, risk perception describes how a person evaluates a prospective flood event in terms of its perceived probability, in other words, the expectation of being exposed to a flood, and severity, that is to say the expected damage if a flood event occurs (Terpstra 2011). The affective subcomponent of threat appraisal refers to fears or worries about flooding and accounts for the effect that emotions have on flood risk behavior (Zaalberg et al. 2009).

According to the PMT, threat appraisal is a key determinant of protection motivation. However, several studies on flood mitigation fail to confirm the expected positive relationship or only find a weak one (see review by Bubeck, Botzen, and Aerts 2012). Poussin, Botzen, and Aerts (2014) for instance, even find a negative relationship between perceived risk of flood damage and mitigation behavior for two out of three mitigation measures. While some studies identify fear as a driver for protective motivation (e.g. Bubeck, Botzen, and Aerts 2012; Kievik and Gutteling 2011; Terpstra 2011), others only find an indirect effect (Zaalberg et al. 2009) or no significant effect at all (Grothmann and Reusswig 2006). According to Bubeck, Botzen, and Aerts (2012) most of the reported effect sizes for fear are relatively small. This is also supported by a recent meta-analysis by Bamberg et al. (2017), who find only small effects of both threat appraisal (*r* = .23) and negative emotions (*r* = .17) on flood preventive behaviors.

#### 2.2.2. Coping appraisal

Coping appraisal refers to the cognitive process by which a person evaluates possible responses that may reduce the perceived threat. Coping appraisal includes the three subcomponents response efficacy, self-efficacy, and response costs (Floyd, Prentice-Dunn, and Rogers 2000; Rogers and Prentice-Dunn, 1997). Response efficacy is defined as a person’s belief that a protective action will be effective in reducing the expected damage from a specific threat. Self-efficacy refers to whether a person feels capable of carrying out this protective action. Response costs capture the financial resources, time, and effort required to implement the action.

Coping appraisal has frequently been reported to have a strong influence on flood mitigation behavior (e.g. Bubeck et al. 2013; Kievik and Gutteling 2011; Grothmann and Reusswig 2006). Bamberg et al. (2017) and Zaalberg et al. (2009) conclude that the correlation between coping appraisal and flood mitigation is stronger than the correlation between threat appraisal and flood mitigation. In general, the PMT also proposes a negative effect of coping appraisal on nonprotective responses (Milne, Sheeran, and Orbell 2000), suggesting that high coping beliefs reduce maladaptive behavior. In the context of private flood mitigation, however, only very few studies report results on this particular relationship (e.g. Richert, Erdlenbruch, and Figuières 2017; Grothmann and Reusswig 2006).

#### 2.2.3. Non-protective responses

Non-protective responses are not effective in reducing the physical risk of flooding, but ‘help to avoid or suppress the negative emotions associated with it’ (Bubeck, Botzen, and Aerts 2012, 3511). According to the PMT, this type of responses lowers protection motivation and therefore inhibits protective behavior (Milne, Sheeran, and Orbell 2000). A number of non-protective responses have been examined in flood mitigation behavior, including denial, fatalism, and wishful thinking and others. Moreover, overreaching and unrealistic reliance on social support and public flood protection may also qualify as non-protective responses, as such reliance can lead to others (rather than oneself) being held responsible for engaging in precautionary behavior (Babcicky and Seebauer 2017; Bubeck et al. 2013).

However, the effect of non-protective responses on protection motivation remains unclear. Previous evidence shows that non-protective responses may indeed reduce protection motivation (e.g. Siegrist and Gutscher 2008; Grothmann and Reusswig 2006). But the negative relationship, as proposed by the PMT, could only be confirmed for some non-protective responses, and they do not seem to have a significant impact on all types of flood mitigation measures. For instance, Richert, Erdlenbruch, and Figuières (2017) and Poussin, Botzen, and Aerts (2014) report the somewhat counter-intuitive result that trust in public flood protection measures makes flood-prone households more likely to take action; this is also supported by Bamberg et al. (2017), who, regrettably, did not include any other non-protective responses in their meta-analysis. Richert, Erdlenbruch, and Figuières (2017, 349) conclude that the effect of trust in public protection ‘is still unclear and requires further investigation’.

The results of previous studies on the relationship between threat appraisal and non-protective responses, however, are more consistent (Richert, Erdlenbruch, and Figuières 2017; Zaalberg et al. 2009; Grothmann and Reusswig 2006). But what is striking is that these studies find a negative relationship, as opposed to theory which suggests a positive association; however, as threat appraisal is presumably influenced by feedback effects over time, having adopted wishful thinking for instance, may reduce the perception of risk. Putting aside the direction of the relationship, there seems to be an empirical consensus that threat appraisal and non-protective responses are significantly related.

### 2.3. The protective and non-protective route

Much of the existing PMT research on private flood mitigation has produced conflicting and inconsistent results. The previous sections have demonstrated that empirical studies report different results for both the direction and significance of the relationships between PMT components. This is surprising, considering that these studies apply the same theoretical perspective (i.e. PMT) to explain the same behavioral domain: flood mitigation by private households.

Nevertheless, the empirical evidence reviewed above suggests that two paths in the PMT framework are substantially more consistent than others: the protective route from coping appraisal to protective behavior, and the non-protective route from threat appraisal to non-protective responses. This is also in line with Roger’s (1983) original notion of the PMT differentiating between two cognitive processes, threat appraisal and coping appraisal. However, the two processes appear to lead to two separate endpoints (either protection motivation or non-protective responses) and they do not seem to interact with each other (i.e. the assumption that the effect of threat appraisal on protection motivation is mediated by non-protective responses). The present study sets out to empirically verify the nature of the two routes across six different private flood mitigation behaviors and four non-protective responses.

This article also aims to improve upon methodological weaknesses of existing work. Several conflicting results from earlier studies may be traced back to methodological designs that could not fully uncover the linkages between the PMT components: (1) Some studies lack important PMT components such as fear (Le Dang et al. 2014) or response costs (Le Dang et al. 2014; Zaalberg et al. 2009). (2) The widespread usage of conjoint measures rules out the possibility of examining PMT components separately. Grothmann and Reusswig (2006) collect data on three non-protective responses, but bracket them together into a single conjoint measure. Zaalberg et al. (2009), Bubeck et al. (2013) and Richert, Erdlenbruch, and Figuières (2017) aggregate a series of protective responses into a single index. (3) The dichotomization of protective responses (e.g. in Bubeck et al. 2013; Grothmann and Reusswig 2006) reduces statistical power and increases the risk of false positives (Altman and Royston 2006). (4) Most importantly, the common use of regression analysis only allows a single dependent variable (i.e. protection motivation) at a time to be explained, whereas the model structure of the PMT consists of two endogenous variables, protection motivation and non-protective responses.

In the light of these shortcomings, we employ structural equation modeling to analyze the interrelations among the PMT components. In the present study, most components and subcomponents are operationalized as latent factors, each consisting of several continuous items, thereby taking measurement error into account. The subcomponents (e.g. cognitive and affective threat appraisal, non-protective responses) are reflected by latent factors, which allow us to examine their respective interrelations simultaneously. The large sample size permits a robust assessment of statistical significance. However, this study is not exempt from the critique of partial coverage of the PMT, as it did not include the subcomponent self-efficacy (see Section 5.2). Nevertheless, we believe that the more rigorous methodological approach used in this study has the potential to reconcile existing findings and to increase the validity of PMT applications in research on flood mitigation behavior.

## 3. Study design and method

### 3.1. Study setting and data collection

Austria was chosen as a case study region due to its long history of extreme flood events (Prettenthaler et al. 2015). In the recent past, severe hydrological events have caused major economic damage across the country. The 2002 flood event resulted in total damage costing €3.2 billion, and financial losses for the 2013 flood are estimated to be around €2 billion (Neunteufel et al. 2015). The successful implementation of Austria’s National Adaptation Strategy is contingent on both planned, public and autonomous, and private adaptation (König et al. 2015). The combination of the country’s vulnerability to flood risks, its history of state governmentality in flood protection, and its recent policy shift to promote private flood adaptation makes Austria a particularly interesting case (BMLFUW 2017; Habersack, Bürgel, and Kanonier 2009).

This paper uses data collected in a cross-sectional questionnaire survey carried out in 10 flood-prone municipalities in Austria between October 2014 and February 2015. Municipalities were selected based on the official natural hazards map for Austria (HORA 2015). Seven municipalities were chosen from the province of Styria and three from the province of Vorarlberg, most of them situated in Alpine mountain regions. While Styria is located in the eastern part of Austria, Vorarlberg is located in the West. This spatial variation helps us to expand the geographical scope of our study and generalize our findings to other, similar regions in Austria. Self-completion questionnaires were distributed as an insert in municipal newspapers. Questionnaires could be returned in a dedicated postage prepaid envelope, dropped off at the local municipal office, or completed in an identical online survey. In the surveyed regions, municipal newspapers are distributed to all residents; therefore, this method allowed us to reach almost the entire study population (i.e. all households living in the 10 municipalities). Respondents were invited to participate in a lottery for gift vouchers upon completing the survey (5 × 30 Euro in each municipality). The head of each household was asked to complete the questionnaire on behalf of the other household members.

A response rate of 12.8% yielded a sample of 2,007 respondents with a fairly equal distribution across the municipalities, except for Lustenau which constitutes 65% of our sample. This is not surprising, as Lustenau is the largest municipality (> 8,000 households) of all those surveyed (500–3,000 households). Table A1 shows that most sample characteristics conform well to the census data of the population, except for male, older and medium-income respondents, who are slightly overrepresented. Among all surveyed households, 26% indicated that they had already experienced a flood event in the past. While 65% do not know whether they live in a flood risk zone, 20% stated that their house is located within a flood risk zone. About 15% indicated that they do not live in a flood risk zone. Engagement in precautionary action appears to be rather low: 35% stated that they are insured against flood risks. This however, typically refers to standard household insurances that also cover minor flood damages. Only 28% of the households have already implemented one or more private mitigation measures besides insurance.

### 3.2. Measures

Most PMT components were specified as latent factors, measured on continuous multi-item scales. In the questionnaire, items were presented in mixed order, so that their conceptual assignment to factors was not transparent to the respondents. Factor and item conceptualization were largely based on existing studies on private flood mitigation. Exact item wordings and descriptive statistics are given in Table A2 (see Appendix). Prior to distribution, the questionnaire was pretested to ensure clarity and relevance of the items. All original items were in German and translated for this paper.

*Protective responses* included six measures in total: coordination with neighbors, emergency plan for all household members, purchase of private flood insurance, no valuable belongings in the basement or on the ground floor, preventive measures (e.g. sandbags, flood barriers), and structural protection at parts of the building (e.g. waterproof doors and windows, oil tank securement). This selection of responses covers a broad spectrum from low- to medium- and high-cost measures (Rözer et al. 2016). Protective responses were measured on a six-step response scale, ranging from already implemented (6), to very likely (5), rather likely (4), rather unlikely (3), very unlikely (2) or not feasible (1).

*Threat appraisal* was measured on both the cognitive (risk perception) and affective (fear) level. With respect to risk perception, respondents were asked to indicate how they assess the probability and potential damages of a severe flood affecting their building within the next 10 years (Grothmann and Reusswig 2006). Answers were measured on a 10-step response scale, ranging from very likely (10) to very unlikely (1) and very high (10) to very low (1), respectively. For the affective subcomponent fear, respondents were asked to indicate their worries about a potential flood event on a 5-step response scale (two items).

*Coping appraisal* was measured on a four-step response scale, adapted from Bubeck et al. (2013). Respondents were asked to rate response efficacy, ranging from very effective (4) to very ineffective (1), and response costs, ranging from very high (4) to very low (1). Both response efficacy and response costs were assessed for each of the six protective responses separately. The two subcomponents were measured as single items, since the protective responses to which they refer to, are very narrow behavioral domains.

Each of the four *non-protective responses* was measured by three items, using a five-step response scale, ranging from fully agree (5) to fully disagree (1). Statements relating to the following four non-protective responses were included in the questionnaire: denial, fatalism, reliance on social support, and reliance on public flood protection. The first two were adapted from Grothmann and Reusswig (2006). Reliance on social support captures the confidence in receiving sufficient external support (i.e. from neighbors, friends, and family) during a flood event. Reliance on public flood protection was measured by asking respondents to indicate their satisfaction with and trust in the public management of flood risks in their municipality (Richert, Erdlenbruch, and Figuières 2017). Although reliance on public flood protection had been used as an explanatory factor in previous studies (e.g. Richert, Erdlenbruch, and Figuières 2017; Poussin, Botzen, and Aerts 2014; Terpstra 2011; Grothmann and Reusswig 2006), it had not yet been explicitly modeled as a non-protective response.

### 3.3. Analytical approach

We apply structural equation modeling to analyze the strength of influence between the PMT components and subcomponents. SEM is a statistical method that combines confirmatory factor analysis and path analysis (Weston and Gore 2006) in order to examine hypothesized causal relationships between latent factors (Byrne 2010). Here, factors comprise risk perception, fear, response efficacy, response costs, protective, and non-protective responses. Each factor is measured by one to three items, where multi-item measurement is the preferred method, as this type of measurement controls for measurement error (Nachtigall et al. 2003; Jaccard and Wan 1995).

All SEM models are calculated in AMOS 23 software with raw data using full information maximum likelihood (FIML) estimation. FIML is considered superior to, for example, listwise deletion with respect to handling missing data (Enders and Bandalos 2001). Since FIML takes the full number of cases into account, all models reported in the results section refer to the full sample size of 2,007 households. It should be noted, however, that case numbers for the variables reported in Table A2 are smaller due to missing values.

Fit indices help to assess how well a model represents the observed data. This study employs the most frequently used model fit indices: the comparative fit index (CFI), the normed fit index (NFI), and the root mean-square error of approximation (RMSEA; Byrne 2010; Bentler 1990). A model is considered acceptable if the CFI and NFI reach a minimum threshold of .90 (Hu and Bentler 1999; Marsh and Hocevar 1985). The common cut-off criterion for the RMSEA is .08 (Browne and Cudeck 1993) and the *χ*^2^/df ratio should not exceed the range of 2–5 (Marsh and Hocevar 1985).

We only discuss standardized path coefficients larger than 0.1 in the following sections. According to Cohen (1992), coefficients between 0.1 and 0.3 are considered small effects. In our study, coefficients that fall below the 0.1 threshold may reach statistical significance due to the large sample size, but are still considered negligible. However, all coefficients are given in Table 2 for reference.

**Table 1.** Intercorrelations of protective and non-protective responses.

	Intercorrelations between protective responses				
	Coordination with neighbors	Emergency household plan	Flood insurance	No valuable belongings basement/ground floor	Preventive measures
Coordination with neighbors	–				
Emergency household plan	.332**	–			
Flood insurance	.117**	.143**	–		
No valuable belongings basement/ground floor	.198**	.201**	.146**	–	
Preventive measures	.377**	.277**	.109**	.119**	–
Structural protection	.345**	.255**	.187**	.257**	.374**
	**Intercorrelations between non-protective responses**				
	Fatalism	Denial	Social support		
Fatalism	–				
Denial	.441**	–			
Social support	.243**	.220**	–		
Public protection	.389**	.385**	.481**		

* p<.05; **p<.01; Intercorrelations between protective responses based on bivariate correlations of single items; *n* = 1,580–1,709; Intercorrelations between non-protective responses based on CFA; *χ*^2^(df) = 172.765(48)**; *χ*^2^/df =3.6; CFI=.984; NFI=.978; RMSEA=.036 (10%-CI=.030–.042); *n* = 2,007.

**Table 2.** SEM model results for six protective and four non-protective responses (each column represents one model; 24 models in total).

	1. Coordination with neighbors				2. Emergency household plan				
PMT paths	Denial	Fatalism	Social support	Public protection	Denial	Fatalism	Social support	Public protection	
Risk perception –> protective response	.06	.04	.08*	.06	−.07	−.07	−.06	−.10*	
Fear –> protective response	.03	.03	.04	.03	.21**	.21**	.21**	.19**	
Response efficacy –> protective response	.51**	.51**	.49**	.51**	.55**	.55**	.55**	.56**	
Response costs –> protective response	−.16**	−.17**	−.16**	−.16**	−.04	−.03	−.04	−.03	
Non-protective response –> protective response	.06*	.00	.14**	.03	−.02	−.03	.00	−.09**	
Risk perception –> non-protective response	−.36**	−.29**	−.27**	−.49**	−.37**	−.29**	−.29**	−.50**	
Fear –> non-protective response	−.08	.21**	−.07	−.09*	−.08	.20**	−.06	−.10*	
Response efficacy –> non-protective response	.01	.07*	.21**	.12**	.02	.10**	.12**	.15**	
Response costs –> non-protective response	−.04	−.01	−.01	−.02	.10**	.09**	.04	.05*	
Chi^2^ (df)	76.7 (28)**	101.8 (28)**	86.0 (28)**	102.0 (28)**	83.0 (28)**	114.5 (28)**	77.1 (28)**	104.6 (28)**	
Chi^2^/df	2.74	3.64	3.07	3.64	2.97	4.09	2.75	3.74	
CFI	.992	.984	.991	.989	.991	.982	.992	.989	
NFI	.987	.978	.987	.985	.986	.976	.988	.985	
RMSEA	.029	.036	.032	.036	.031	.039	.030	.037	
10%-CI RMSEA	.022-.037	.029-.044	.025-.040	.029-.044	.024-.039	.032-.047	.022-.038	.03-.045	
SMC for protective response	.298	.295	.312	.296	.333	.333	.333	.336	
SMC for non-protective response	.184	.045	.144	.328	.199	.059	.124	.348	
	3. Flood insurance				4. No valuable belongings basement/ground floor				
PMT paths	Denial	Fatalism	Social support	Public protection	Denial	Fatalism	Social support	Public protection	
Risk perception –> protective response	.03	.05	.05	.02	.02	.01	.00	.00	
Fear –> protective response	.08*	.10*	.09*	.08*	.06	.03	.05	.04	
Response efficacy –> protective response	.38**	.39**	.38**	.40**	.38**	.37**	.37**	.37**	
Response costs –> protective response	−.20**	−.20**	−.20**	−.20**	−.41**	−.41**	−.41**	−.41**	
Non-protective response –> protective response	−.07*	−.03	−.01	−.08**	.09**	.08**	.03	.01	
Risk perception –> non-protective response	−.37**	−.28**	−.28**	−.49**	−.37**	−.29**	−.28**	−.50**	
Fear –> non-protective response	−.08	.21**	−.04	−.08	−.08	.22**	−.03	−.07	
Response efficacy –> non-protective response	.03	.18**	.16**	.19**	−.03	.06*	.08**	.08**	
Response costs –> non-protective response	.03	.04	−.03	.02	−.03	−.03	−.06*	−.04	
Chi^2^ (df)	96.1 (28)**	100.4 (28)**	78.3 (28)**	105.6 (28)**	141.5 (28)**	150.0 (28)**	127.6 (28)**	159.9 (28)**	
Chi^2^/df	3.43	3.58	2.80	3.77	5.05	5.36	4.56	5.71	
CFI	.988	.984	.992	.988	.981	.974	.985	.981	
NFI	.983	.978	.988	.984	.976	.968	.981	.977	
RMSEA	.035	.036	.030	.037	.045	.047	.042	.048	
10%−CI RMSEA	.027−.043	.028−.044	.022−.038	.030−.045	.038−.052	.039-.054	.035–.050	.041–.056	
SMC for protective response	.210	.206	.206	.209	.314	.312	.308	.308	
SMC for non-protective response	.186	.070	.120	.342	.184	.045	.106	.317	
	5. Preventive measures				6. Structural protection				
PMT paths	Denial	Fatalism	Social support	Public protection	Denial	Fatalism	Social support	Public protection	
Risk perception –> protective response	.08	.05	.09*	.02	−.03	−.04	.00	−.04	
Fear –> protective response	.09*	.10*	.09*	.07	.09*	.09*	.09*	.07	
Response efficacy –> protective response	.45**	.45**	.44**	.46**	.31**	.31**	.31**	.31**	
Response costs –> protective response	−.17**	−.17**	−.17**	−.17**	−.27**	−.27**	−.27**	−.27**	
Non-protective response –> protective response	.02	−.09**	.06*	−.11**	.00	−.04	.07**	−.04	
Risk perception –> non-protective response	−.37**	−.29**	−.28**	−.50**	−.36**	−.29**	−.28**	−.50**	
Fear –> non-protective response	−.08	.21**	−.05	−.09*	−.08	.22**	−.05	−.08*	
Response efficacy –> non-protective response	.02	.06*	.12**	.13**	−.03	−.07*	.07**	.04	
Response costs –> non-protective response	.00	.05	−.03	.00	−.08**	−.04	−.03	−.08**	
Chi^2^ (df)	80.8 (28)**	99.0 (28)**	67.4 (28)**	92.2 (28)**	161.5 (28)**	175.8 (28)**	162.9 (28)**	175.4 (28)**	
Chi^2^/df	2.89	3.54	2.41	3.29	5.77	6.28	5.82	6.27	
CFI	.991	.984	.994	.990	.976	.966	.978	.977	
NFI	.986	.978	.989	.986	.971	.961	.974	.973	
RMSEA	.031	.036	.026	.034	.049	.051	.049	.051	
10%-CI RMSEA	.023–.039	.028–.043	.018–.035	.026–.042	.042–.056	.044–.059	.042–.056	.044–.059	
SMC for protective response	.250	.256	.253	.256	.172	.174	.177	.173	
SMC for non-protective response	.184	.047	.117	.332	.187	.048	.108	.320	

* p<.05; **p<.01; standardized path coefficients and correlations; SMC = squared multiple correlation; correlation between risk perception and fear = .71–.72**; *n* = 2,007.

## 4. Results and discussion

### 4.1. Discriminant validity of protective and non-protective responses

Discriminant validity is established if conceptually distinct factors are confirmed as unrelated or just moderately related to each other. To examine the discriminant validity of our dependent variables, we calculate intercorrelations for all protective and non-protective responses. For the six single-item protective response measures, bivariate Pearson correlations are calculated. Since the four non-protective responses are operationalized as latent factors, intercorrelations are derived from a confirmatory factor analysis (CFA).

Results for the intercorrelations of protective and non-protective responses are given in Table 1. Generally, we find low to moderate correlations between protective responses, ranging from *r* = .109 between flood insurance and preventive measures to *r* = .377 between coordination with neighbors and preventive measures. Similarly, correlations between non-protective responses range from *ϕ* = .220 between denial and reliance on social support to *ϕ* = .481 between reliance on social support and public protection.

The low to moderate correlations imply that most protective responses are implemented independently from each other and that households distinguish between different non-protective responses. Since the six protective and four non-protective responses are conceptually distinct and feature good discriminant validity, we do not aggregate them into compound measures. An aggregation of measures, as applied in Grothmann and Reusswig (2006), bears the risk of lumping together different risk attitudes and behaviors and overly simplifying the dynamics of households’ decision making. Instead, we estimate separate SEM models for all 24 combinations of protective and non-protective responses. This approach allows us to fully disentangle the protective and non-protective pathways over the entire range of six protective and four non-protective responses.

### 4.2. Model structure and results

We test the relationships between the PMT components and subcomponents, as hypothesized by the theory, in 24 SEM models. Each model has the same model structure, as shown in Figure 1.

All SEM results are given in Table 2, where each sub-table (indicated by numbers 1–6) represents a single protective response for which four separate models (one for each non-protective response) are calculated. In each sub-table, the upper nine rows represent the paths as hypothesized by the PMT. Therefore, the sub-tables read as follows: For example, the row ‘Risk perception –> protective response’ in sub-Table 1 gives the path coefficient for the effect of risk perception on the protective response ‘coordination with neighbors’, considering a specific non-protective response as represented by the respective column (e.g. denial in column 1). In the lower rows, model-specific fit indices are given.

The fit indices obtained indicate a good to excellent fit for all 24 models, with the only exception being the *χ*^2^/df ratio, which slightly exceeds the acceptable range in three models on not keeping valuable belongings in the basement or on the ground floor, and in the models on structural protection. The quality of the measurement model (i.e. how well the factors are represented by their assigned items) is reflected both in the good overall model fit and in the individual factor loadings (all loadings >.60, except for one fatalism item with a loading of .57; see Table A2). According to the squared multiple correlations (SMC), our models explain between 17% and 34% of the variance in protection motivation and between 5% and 35% of the variance in non-protective responses.

As an aid to reading Table 2, let us use interpretation of the results for the protective response ‘flood insurance’ as an illustration. The path coefficients in the first five rows of Table A1 describe the protective route: The association between threat appraisal and the motivation to purchase flood insurance seems to be almost nonexistent. The motivation to purchase insurance does not seem to be predicted by non-protective responses, either, with no path coefficient surpassing the 0.1 threshold for small effect sizes. However, coping appraisal is closely and positively related to the motivation to purchase flood insurance: the higher the response efficacy (standardized coefficients ranging between .38 and .40 across the four models) and the lower the response costs (−.20 in all models), the greater the intention to purchase flood insurance. The last four rows refer to the non-protective route. The path coefficients show that non-protective responses are primarily driven by risk perception—the lower the perceived risk, the higher the tendency to adopt non-protective responses (standardized coefficients ranging from −.28 to −.49); this effect is most pronounced for reliance on public protection. Similar to risk perception, fear is negatively related to non-protective responses, but the effect is weaker throughout the models. The only exception is fatalism, which is found to increase with levels of fear (.21–.22). Compared to threat appraisal, the subcomponents of coping appraisal (response efficacy and response costs) show weaker effects on non-protective responses throughout, with high levels of response efficacy (with regard to flood insurance) leading to higher levels of fatalism, reliance on social support and public protection (.16 to .19).

### 4.3. Determinants of the protective route

Contrary to what the PMT suggests, this study does not find a consistent association between threat appraisal and protection motivation. The only effect of threat appraisal on protection motivation found is for the subcomponent fear, prompting emergency plans for household members. Apparently, fear may spur people to action when it comes to last-minute evacuation procedures in an impending emergency, but fear fails to promote long-term, planned, precautionary measures. Also, risk perception only presents a marginal impact in one of the four models on having an emergency household plan, but the coefficient barely exceeds the 0.1 threshold for small effect sizes.

If threat appraisal does not affect protection motivation directly, there might be an indirect effect through non-protective responses. However, this mediating role of non-protective responses is not supported by our results either, which refute a consistent association between non-protective responses and protection motivation. Nonetheless, a weak relationship is found between reliance on social support and coordination with neighbors. Households which assume that they will receive social support during a flood event are more likely to coordinate with neighbors, presumably because neighbors are the most proximal network that could provide social support in a flood event. Another weak relationship is found between reliance on public protection and preventive measures. Households which assume that they will be protected by public flood mitigation are less likely to take preventive measures such as storing sandbags.

Yet, we find strong evidence for the protective route, that is to say the impact of coping appraisal on protective responses. Of the two subcomponents of coping appraisal investigated here, response efficacy is identified as the strongest predictor of protective responses. This is particularly evident with low-cost responses, such as coordination with neighbors and an emergency plan for household members, where the assessment of the effectiveness of a response clearly overshadows the influence of its costs. In the case of medium to high-cost responses (e.g. structural protection), on the other hand, both response efficacy and response costs are relevant. Response costs are identified as significant predictors of not keeping valuable belongings in the basement/ground floor and structural protection.

### 4.4. Determinants of the non-protective route

The model results outlined in Table 2 show that non-protective responses are almost uniformly related to threat appraisal across the 24 models. The most relevant driver of non-protective responses is risk perception, the cognitive subcomponent of threat appraisal. Risk perception has a consistently negative relationship with all four non-protective responses. According to the PMT, the relationship is expected to be positive, as overwhelming risk makes people avoid the negative emotions and the responsibility associated with it (Milne, Sheeran, and Orbell 2000). However, the negative sign in our models is in line with previous studies on private flood mitigation behavior (Zaalberg et al. 2009; Grothmann and Reusswig 2006). Possibly, the negative sign is a result of a gradual process of self-assurance: Once a person has started to deny a threat, risk perception goes down, otherwise the non-protective response would be ineffective. After some time has passed, low-risk perception has developed, together with higher levels of non-protective responses, yielding a negative relationship. Since residents in flood-prone areas have usually lived with the risk for some time, cross-sectional surveys can only observe the endpoint of this process. In the present study, the strongest negative association is found between risk perception and reliance on public protection, a finding which is in line with Terpstra (2011). Households which perceive flood risks to be low tend to rely more on public protection. Again, when assuming that the relationship goes in the opposite direction, the sign seems plausible: If households trust in a public narrative that the municipality is sufficiently protected against flood risks, those households may indulge in a false feeling of security and tend to downplay their personal risk (Babcicky and Seebauer 2017).

Fear plays a less important role. Similar to risk perception, fear is generally negatively related to non-protective responses, except for the association with fatalism. The positive relationship between fear and fatalism suggests that households which suffer from fear of flooding also tend to be more fatalistic. This could be explained by a feedback loop similar to a downward spiral: Fatalism refers to the belief that events are predetermined, which in turn could increase fear. Households which feel powerless may even become more afraid of flooding, because they are exposed to a threat they cannot control.

The effect of coping appraisal on non-protective responses is small and almost negligible, which implies that the non-protective route is separated from coping beliefs. However, there is evidence that response efficacy increases reliance on social support and public protection to some extent, particularly in the models on coordination with neighbors, emergency household plan, flood insurance, and preventive measures. What these responses have in common is that their successful implementation depends on cooperation with other individuals (e.g. neighbors, household members), companies (e.g. insurance providers) or civic actors (e.g. emergency volunteers helping with sandbagging). This may explain why households which assess these responses as effective are also more likely to rely on social support or public protection.

## 5. Conclusion

To our knowledge, this study is the first attempt to apply a comprehensive version of the PMT to private flood mitigation by means of structural equation modeling. This methodological approach allows us to disentangle the interrelations between the PMT components and subcomponents, and thus provide more nuanced insights into which factors drive or impede flood mitigation. Our results clearly show two separate routes within the PMT: Whereas coping appraisal is strongly associated with protection motivation, threat appraisal is closely linked to non-protective responses. The effect of non-protective responses on protection motivation is negligibly small. The validity of our results is further sustained by the fact that the pattern of two separate routes is observed consistently across all combinations of six protective and four non-protective responses.

Overall, the theoretical assumption that non-protective responses take a mediating role in the PMT framework is not supported. Rather, it seems that non-protective responses represent just another endpoint, as is the case with protective responses. Flood-prone households may take the protective route, which is dominated by coping beliefs, or the non-protective route, which is driven by perceived flood risk. However, apparently, taking one route does not make a household more or less likely to take the other route.

### 5.1. Implications for flood risk management and risk communication

Our findings have important implications for future flood risk policies and in particular for persuasive communication. If flood risk management aims to motivate private actors to take protective action, risk communication measures should specifically target the protective route and avoid (accidentally) providing incentives that fall within the non-protective route. The results obtained in this study can help guide flood risk management in identifying and taking appropriate actions that will stimulate private precautionary behavior.

According to our results, communication of flood risks alone is not effective in triggering protective behavior. Instead, promoting risk awareness is more likely to evoke justifications for inaction, making households externalize responsibilities to other actors. Among the various non-protectiveresponses, reliance on public protection and social support can be particularly problematic, since households’ expectations regarding the efficacy and capacity of public measures and social support might be largely hypothetical, if they have not been tried and tested during actual flood events. These unrealistic expectations can easily lead to a false sense of security among flood-prone residents. Public dams are usually designed to protect against flood events up to a certain return period; public damage compensation is capped to a certain amount; social support during flood events may be limited because of lack of commitment, or if the number of flood-affected households exceeds the social capacity of the community. Therefore, it is important for risk communication to be transparent as to what extent public protection measures and social support can be effective, acknowledging that residual risk will exist, which then needs to be managed or accepted by the affected population.

Raising awareness of residual risk should be complemented by information on the efficacy and costs of private measures. There are several ways of distributing such information. One is to publish detailed information on the cost-effectiveness of mitigation measures, and another option is to create anecdotal messages, highlighting successful implementations of private measures. Local authorities who are responsible for risk reduction could organize demonstrations or fairs, showcasing the usage and effectiveness of mitigation measures. Also, using individualized photorealistic visualizations of how measures could help safeguard one’s own building could lead to a better understanding of the strengths and weaknesses of different measures. Indirectly experiencing how other households cope with flood risk could also empower households to take action themselves. Engagement as a volunteer worker in flood recovery in other regions, site visits to flood-affected buildings, or informal meetings with flood victims could offer hands-on insights into which measures were or would have been effective in mitigating the impact of flooding. Also, community-based approaches seem promising to promote flood mitigation among participating households. The installation of flood wardens and flood action groups, for instance, can serve as a platform to exchange experiences with regard to the effectiveness and costs of protective measures, or to jointly coordinate measures between adjacent buildings.

Given that more affordable measures are more likely to be implemented, it also seems important to remove financial barriers. Here, subsidies or government loans could play a substantial role, particularly in making high-cost measures such as retrofitting building envelopes more affordable. In some instances, it may be more efficient to use public budgets to increase the resilience of selected flood-prone buildings, than to erect structural flood defenses for entire catchment areas. But there are also actions that can be taken by private households directly. One such option is to create private purchasing pools, allowing households to negotiate discounts when buying flood mitigation equipment or building materials in bulk. Insurance providers may offer lower flood insurance premiums to customers who invest in property-level flood protection, or may allow (partial) deduction of mitigation expenses from insurance premiums. Another approach to make mitigation more available and affordable relates to market competition. Over time, a growing number of flood mitigation products will naturally result in greater competition and, subsequently, lower prices. Here, governments could systematically promote innovative businesses and remove entry barriers, for example, by streamlining technical and legal standards or licensing procedures.

### 5.2. Limitations and future directions

This study relies on cross-sectional data to analyze the interrelations between the PMT components. Longitudinal data or experimental designs could clarify the direction of influence between risk perception and non-protective responses or between fear and fatalism (see Section 4.4). In line with our results, Rippetoe and Rogers (1987), although in the health domain, confirmed experimentally that high levels of perceived vulnerability and fear were only associated with maladaptive coping (i.e. non-protective responses) but not with adaptive coping (i.e. protective responses).

Still, even in a cross-sectional dataset as used here, we would expect two PMT components to correlate at least moderately if they are theorized to affect each other. Yet, as we find only negligibly small or nonexistent interrelations between the components of the protective and the non-protective route, it appears that these routes might not share a causal connection. Bubeck and Botzen (2013) counter-argue that cross-sectional studies might mask feedback effects between threat appraisal and protection motivation: If some households have already carried out a mitigation measure, this may have decreased their threat appraisal, thereby attenuating the overall correlation between threat appraisal and protection motivation. Yet, only the minority of the households in our sample has actively implemented mitigation measures (see Section 3.1). Therefore, it seems unlikely that this feedback effect is strong enough to statistically compensate a potential correlation between threat appraisal and protection motivation in the majority of the sample.

Another potential blind spot of cross-sectional studies and thereby a promising direction for future research is the stage-driven nature of decision-making. The non-protective route might be more relevant during early decision stages when households become aware about flood risk and contemplate taking action; the protective route might come into play as soon as households actually prepare, implement and maintain mitigation measures. Plotnikoff and Trinh (2010) applied a stage model approach (i.e. Transtheoretical Model) in the health domain and showed that the influence of threat and coping appraisal varies depending on the decision stage.

Our SEM analysis allowed us to address potential mediator effects, for instance that the effect of threat appraisal on protection motivation might be conveyed via non-protective responses (which we find to not be the case). However, the PMT also proposes moderator effects, for example that the combination of high threat and low coping appraisal leads to non-protective responses. Ideally, this multiplicative relationship would be examined using moderation analysis in SEM, or by tracing individuals over time as they develop their appraisals and decide on a course of action or inaction.

This study did not include self-efficacy as a subcomponent of coping appraisal besides response efficacy and response costs. Pretest respondents struggled to distinguish their capability (self-efficacy, ‘can do’ according to Bandura 2006) from their likelihood (protective response, ‘will do’) to implement a specific protective measure; similarly, Zaalberg et al. (2009) report multicollinearity between coping appraisal subcomponents. In contrast, self-efficacy appears as a clear and unique construct in Bubeck et al. (2017) and Richert, Erdlenbruch, and Figuières (2017). Therefore, as we opted to minimize response ambiguity, it remains to be confirmed in future studies whether self-efficacy is also separated from the non-protective route. Still, the fact that one subcomponent was left out of the coping appraisal construct does not invalidate the empirical findings regarding each of the other two subcomponents.

Despite these limitations, our analysis of the cognitive processes involved in decision making of flood-prone households revealed a clear and consistent pattern of two separate routes. Further studies are required to verify whether this pattern also holds for other hazards and in other cultural contexts.

## Appendix

**Table A1.** Sample characteristics.

Municipality		Percentage	Gender	Age (years)					Monthly net household income (€)						Flood experience	Risk zone		
		of total sample	Female	20–34	35–49	50–64	65–79	≥80	<1,100	1,100–1,599	1,600–2,599	2,600–3,999	4,000–5,500	>5,500	Yes	Yes	No	Don't know
Eisenerz	SD	3.1%	40%	9%	17%	31%	39%	5%	11%	30%	39%	11%	2%	7%	18%	25%	32%	43%
	PD	n.a.	53%	12%	18%	27%	29%	14%	7%	14%	21%	34%	17%	7%	n.a.	10–30%	n.a.	n.a.
Fernitz	SD	2.2%	25%	11%	46%	27%	16%	0%	10%	10%	17%	43%	17%	3%	63%	23%	8%	70%
	PD	n.a.	52%	22%	30%	28%	14%	6%	7%	14%	21%	34%	17%	7%	n.a.	10–30%	n.a.	n.a.
Gosdorf	SD	3.7%	51%	6%	30%	36%	20%	7%	14%	32%	36%	16%	2%	0%	49%	28%	24%	48%
	PD	n.a.	50%	20%	28%	27%	18%	6%	7%	14%	21%	34%	17%	7%	n.a.	10–30%	n.a.	n.a.
Gössendorf	SD	7.6%	34%	13%	37%	32%	17%	1%	1%	17%	37%	32%	10%	4%	68%	44%	5%	52%
	PD	n.a.	51%	22%	32%	25%	15%	6%	7%	14%	21%	34%	17%	7%	n.a.	0–10%	n.a.	n.a.
Hatzendorf	SD	2.9%	29%	18%	32%	32%	18%	0%	13%	27%	31%	22%	4%	2%	50%	22%	29%	49%
	PD	n.a.	50%	23%	28%	27%	16%	6%	7%	14%	21%	34%	17%	7%	n.a.	0–10%	n.a.	n.a.
Mooskirchen	SD	2.5%	54%	17%	28%	37%	17%	0%	13%	10%	43%	20%	13%	0%	48%	22%	33%	46%
	PD	n.a.	51%	23%	29%	26%	14%	7%	7%	14%	21%	34%	17%	7%	n.a.	10–30%	n.a.	n.a.
Radmer	SD	1.7%	46%	6%	27%	36%	18%	12%	12%	54%	19%	12%	4%	0%	61%	42%	13%	45%
	PD	n.a.	50%	13%	22%	29%	23%	13%	7%	14%	21%	34%	17%	7%	n.a.	10–30%	n.a.	n.a.
Lustenau	SD	65.0%	34%	12%	25%	29%	26%	8%	6%	15%	38%	29%	9%	3%	15%	16%	9%	75%
	PD	n.a.	51%	27%	29%	24%	16%	5%	8%	10%	36%	31%	8%	7%	n.a.	0–10%	n.a.	n.a.
Mellau	SD	3.1%	38%	8%	28%	34%	18%	12%	4%	20%	41%	26%	7%	2%	59%	20%	21%	59%
	PD	n.a.	49%	26%	28%	24%	15%	6%	3%	9%	24%	35%	22%	7%	n.a.	0–10%	n.a.	n.a.
Nenzing	SD	8.2%	29%	7%	34%	32%	26%	2%	6%	17%	33%	33%	9%	3%	34%	10%	50%	40%
	PD	n.a.	50%	24%	30%	25%	16%	5%	3%	9%	24%	35%	22%	7%	n.a.	10–30%	n.a.	n.a.
All regions	SD	100%	34%	11%	27%	31%	24%	6%	6%	18%	36%	28%	8%	3%	26%	20%	15%	65%
	PD	n.a.	51%	23%	28%	25%	17%	6%	7%	12%	28%	33%	14%	7%	n.a.	n.a.	n.a.	n.a.

SD = sample data; PD = population data; risk zone = flood return period of 300 years or less, or yellow/red risk zone; gender and age data: Statistics Austria (2014); household income data: Statistics Austria (2009); risk zone data: HORA (2015); percentages may not total 100 due to rounding.

**Table A2.** Descriptive statistics, factor loadings and item wordings.

Factor	Item	Response scale	N	Mean	SD	Std. factor loading
Risk perception	How likely do you consider a severe flood to occur at your building within the next ten years?	Ten-step, 10 = very likely	1,934	4.2	2.8	.86
	What level of damage do you expect, if a severe flood occurs at your building?	Ten-step, 10 = very high	1,894	6.0	3.0	.69
Fear	I am afraid of a potential flood.	Five-step, 5 = fully agree	1,951	3.2	1.3	.89
	I am very worried about the potential threat of flooding.		1,867	3.1	1.2	.88
Denial	Most people consider flooding a bigger problem than it actually is	Five-step, 5 = fully agree	1,900	2.4	1.1	.61
	Generally, the current flood hazard is being exaggerated.		1,942	2.3	1.1	.81
	The public discourse overstates the actual flood hazard.		1,862	2.4	1.0	.80
Fatalism	A flood is just an uncontrollable act of nature.	Five-step, 5 = fully agree	1,909	3.9	1.2	.63
	Ultimately, it is an act of fate whether someone is hit by a flood.		1,930	3.0	1.3	.61
	Besides all human causes, all floods are also an act of the powers that be.		1,866	3.7	1.2	.57
Reliance on social support	In a flood event, I can count on support by others.	Five-step, 5 = fully agree	1,901	3.9	1.0	.62
	Many people would help me during a flood.		1,931	3.3	1.0	.86
	In a flood event, many people would stand by me.		1,860	3.4	1.0	.89
Reliance on public protection	Thanks to public protection, I feel protected from a potential flood.	Five-step, 5 = fully agree	1,910	3.5	1.2	.78
	I can entirely rely on public protection in my municipality.		1,949	3.5	1.1	.78
	Public flood protection makes me feel safe.		1,945	3.5	1.1	.82
Protective response	How likely are you to take the following flood protection measures?					
	Coordination with neighbors (e.g. joint emergency plan, joint structural measures)	Six-step, 6 = already implemented, 5 = very likely, 2 = very unlikely, 1 = not feasible	1,694	3.0	1.4	n.a.
	Emergency plan for all household members		1,642	3.9	1.3	n.a.
	Purchase of private flood insurance		1,685	4.2	1.6	n.a.
	No valuable belongings in the basement or on the ground floor		1,641	3.5	1.8	n.a.
	Preventive measures (e.g. sandbags, flood barriers for doors and windows, silicone sealing)		1,709	3.6	1.4	n.a.
	Structural protection at parts of the building (e.g. waterproof doors and windows, electrical installations above water level, oil tank / fuel store securement, pressure flap on sewer pipe)		1,693	2.8	1.5	n.a.
Response efficacy	How effective do you rate the following measures in protecting your home from flooding?					
	Coordination with neighbors (e.g. joint emergency plan, joint structural measures)	Four-step, 4 = very effective	1,690	2.7	.9	n.a.
	Emergency plan for all household members		1,714	3.1	.9	n.a.
	Purchase of private flood insurance		1,802	3.0	1.0	n.a.
	No valuable belongings in the basement or on the ground floor		1,696	3.0	1.0	n.a.
	Preventive measures (e.g. sandbags, flood barriers for doors and windows, silicone sealing)		1,721	3.0	.8	n.a.
	Structural protection at parts of the building (e.g. waterproof doors and windows, electrical installations above water level, oil tank / fuel store securement, pressure flap on sewer pipe)		1,676	3.2	.9	n.a.
Response costs	How do you rate the costs (money, time and effort) associated with the implementation of the following measures?					
	Coordination with neighbors (e.g. joint emergency plan, joint structural measures)	Four-step, 4 = very high	1,601	2.5	1.0	n.a.
	Emergency plan for all household members		1,617	2.1	.9	n.a.
	Purchase of private flood insurance		1,629	2.6	1.0	n.a.
	No valuable belongings in the basement or on the ground floor		1,632	2.5	1.0	n.a.
	Preventive measures (e.g. sandbags, flood barriers for doors and windows, silicone sealing)		1,626	3.3	.8	n.a.
	Structural protection at parts of the building (e.g. waterproof doors and windows, electrical installations above water level, oil tank / fuel store securement, pressure flap on sewer pipe)		1,645	2.7	.8	n.a.

N = number of cases; SD = standard deviation; factor loadings refer to the model on purchasing private flood insurance (protective response) and denial (non-protective response); loadings of all other models deviate by no more than ± .02.
